# Supplementary material for: The visual word form area (VWFA) is part of both language and attention circuitry
Source: Nat Commun. 2019 Dec 6;10:5601. doi: 10.1038/s41467-019-13634-z (PMC6898452; doi:10.1038/s41467-019-13634-z)
Supplement: Supplementary file 1 — Supplementary Information [file 41467_2019_13634_MOESM1_ESM.pdf]

Supplementary Information

The visual word form area (VWFA) is part of both language and attention circuitry

Lang Chen et al.

List of Contents

Supplementary Methods

Supplementary Discussions

Supplementary Figures S1-3

Supplementary Tables S1-15

Supplementary Reference

Supplemental Methods

Description of the open-source fMRI datasets

Two datasets were acquired from OpenNeuro to assess task-based activation of vOT and connectivity between VWFA and language and attention networks using the same ROIs employed in our structural and intrinsic functional analyses. We chose these two datasets because: (i) they enabled us to test specific hypotheses regarding differential patterns of connectivity between VWFA and nodes of the language and attention networks during distinct cognitive processes, including word reading and visuospatial attention; (ii) the age range of the participants is comparable to the HCP dataset used for the intrinsic functional and structural connectivity analyses; and (iii) these two studies employed relatively simple task design, which enabled standard contrasts for VWFA task connectivity analysis.

Dataset 1: Word Reading Task. (https://openneuro.org/datasets/ds000003/versions/00001). This dataset was collected from 13 participants (8 males; age range: 18-38 years old) using a block design. Each subject completed a rhyme judgement task with visually-presented words in the scanner. There was a total of 4 runs with words and 4 runs with pseudowords which were interleaved with 7 rest runs. The duration of each run was 5 minutes 20 seconds. In each task run, a total of 8 stimuli were presented, and each word or pseudoword was displayed for 2 seconds with a 0.5 second interval between trials. Details of the data acquisition parameters listed in OpenNeuro were limited, however the TR = 2 sec. This dataset was submitted by G., Xue, and R. A., Poldrack and was supported by the OpenfMRI project and NSF Grant (OCI-1131441) to R. Poldrack. No previous publications were associated with this dataset.

Dataset 2: Visual Flanker Task. (https://openneuro.org/datasets/ds000102/versions/00001). This dataset was collected from 26 participants (16 males; age range: 19.65 – 49.79 years old) using an event-related design. Each participants performed two 5-minute blocks, each containing 12 congruent and 12 incongruent trials, presented in a pseudorandom order. On each trial, participants used one of two buttons on a response pad to indicate the direction of a central arrow in an array of 5 arrows. In congruent trials the flanking arrows pointed in the same direction as the central arrow (e.g., < < < < <), while in more demanding incongruent trials the flanking arrows pointed in the opposite direction (e.g., < < > < <). The stimuli was displayed for 1.5 seconds, and the inter-trial interval (ITI) varied between 8 s and 14 s (mean ITI=12 s). Functional imaging data were acquired using a research dedicated Siemens Allegra 3.0 T scanner, with a standard Siemens head coil, located at the NYU Center for Brain Imaging. 146 contiguous, whole-brain functional volumes were acquired using an echo planar imaging (EPI) sequence (TR=2000 ms; TE=30 ms; flip angle=80, 40 slices, matrix=64x64; FOV=192 mm; acquisition voxel size=3x3x4mm) during each of the two flanker task blocks. Additional details of this dataset have been published previously[1–3].

fMRI data preprocessing and analysis

<u>Preprocessing:</u> Data were analyzed using a general linear model implemented in the Statistical Parametric Mapping program (SPM12[4]; Wellcome Trust Centre for Neuroimaging, London, United Kingdom). Images were realigned to correct for movement, denoised, spatially

normalized to Montreal Neurological Institute (MNI) space, and smoothed with an effective Gaussian kernel of 6 mm.

Voxelwise analysis of fMRI activation: Task-related brain activation in the block-design data from Dataset 1 and the event-related design data from Dataset 2 were first modeled at the individual subject level using the general linear model (GLM) implemented in SPM12. Interpolated volumes flagged at the preprocessing stage were deweighted in the individual subject analysis. For Dataset 1, voxel-wise t-statistics maps contrasting the task blocks to the rest blocks (i.e., [word – rest] and [pseudoword – rest] contrasts) were generated for each participant. For Dataset 2, trials associated with correct responses were isolated from those associated with incorrect response, and individual-level activation maps were generated for [correct congruent – rest] and [correct incongruent – rest] contrasts. At the group level, our analysis focused on the [word – rest] conditions for Dataset 1 (reading) given our specific interest in activity profiles during word reading. For Dataset 2 (flanker), our group-level analysis focused on the [correct incongruent – rest] condition to enable a focus on activity profiles during a challenging visuospatial attention processing task. We corrected for multiple comparisons at the cluster level by using Monte Carlo simulations implemented in Matlab. Consistent with recent recommendations for reporting fMRI activation[5], we used a conservative height threshold $p < .001$ with a cluster extent threshold of 41 voxels ($p < .01$).

Functional connectivity analysis: We used a generalized form of Psychophysiological Interaction (gPPI)[6] as implemented in the "Generalized Form of Context-Dependent Psychophysiological Interactions" SPM toolbox. Consistent with intrinsic functional and structural connectivity analyses, a 6-mm sphere centered at (-45, -57, -12) served as the VWFA seed for the functional connectivity analysis. All target ROIs in the language and attention networks were identical to those used in the intrinsic functional and structural connectivity analyses (**Table S3**). At the individual participant level, we included: (i) regressors for the psychological variables (e.g., in Dataset 1, two conditions: word and pseudoword); (ii) one regressor for the physiological variable (i.e., the time course of the seed region VWFA); and (iii) regressors for the psychophysiological interaction term (i.e., the cross-product of each psychological variable with the seed region time course). Movement parameters (x, y, z, roll, pitch, yaw) and a constant term were also included in the model.

Contrast images corresponding to PPI effects (i.e., the [word – rest] contrast in Dataset 1 and the [correct incongruent – rest] contrast in Dataset 2) at the individual level were then entered into a group level statistical analysis. Consistent with the voxelwise analysis of fMRI activation, whole-brain gPPI results were thresholded at a height threshold of $p < .001$ with a cluster extent threshold of 41 voxels ($p < .01$).

The ROI-based analysis was based on the seed-ROI matrix for each participant. At the individual level, the off-diagonal values were averaged for each pair of links (e.g., the VWFA-IFG and IFG-VWFA link values were averaged), and then a one-sample t-test against a mean of zero was used to infer whether there is a significant seed-ROI functional connectivity across the sample. We applied FDR correction on the p-values to correct for multiple comparison. This process was performed separately for Datasets 1 and 2 to examine the VWFA functional connectivity to language and attention networks during the word reading and flanker tasks.

Supplementary Discussions

VWFA is engaged by both word reading and visuospatial attention tasks

Group results from the voxelwise analysis of fMRI activation for the [word – rest] contrast from the reading task revealed significant activation in several key regions implicated in word reading[7–9] including VWFA and posterior FG of ventral occipital cortex, as well as inferior frontal gyrus (IFG) and superior temporal gyrus (STG; Figure S2, green). Additionally, word reading elicited significant activation in bilateral IPS/IPL. Group results for the [correct incongruent – rest] contrast from the visuospatial attention task revealed activation of the frontoparietal attention network[10–12], including dorsolateral PFC, SPL, and IPS (Figure S2, red). Significant activation for this contrast was also evident in ventral occipital regions, including VWFA and pFG. Importantly, voxelwise analysis of fMRI activation showed extensive overlap between word reading and visuospatial attention tasks in ventral occipital regions, including the VWFA region that was used for seed-based structural and intrinsic functional connectivity analyses (purple) and bilateral pFG (yellow). These findings are consistent with previous reports showing VWFA activation for a broad range of visual tasks[13–15] and highlight the functional significance for the precise VWFA region that formed the basis for our structural and intrinsic functional analyses.

VWFA is functionally connected to both language and attention networks during rhyming and visuospatial attention tasks

Consistent with the multiplex model of VWFA function, ROI-based functional connectivity analysis revealed significant connectivity between VWFA and both language and attention networks during word reading and visuospatial attention tasks. Specifically, gPPI results for the [word – rest] contrast of the word reading task showed significant connectivity between the VWFA and key nodes of the language network, including left-hemisphere IFG, SMG, mSTS, and pSTS (Table S12). Moreover, gPPI results for this contrast revealed significant connectivity between VWFA and nodes of the frontoparietal attention network, including left-hemisphere anterior and posterior IPS and MT+.

gPPI results for the [correct incongruent – rest] contrast of the flanker task showed significant connectivity between VWFA and nodes of all nodes of the frontoparietal attention network, including left-hemisphere FEF, anterior and posterior IPS, and MT+ (Table S13). However, interestingly and surprisingly, we also observed significant task-based functional connectivity between VWFA and the language network during this visuospatial attention task, including IFG, SMG, aSTS, and mSTS.

Taken together, results from task-based functional connectivity analyses reveal that: (1) the VWFA was strongly activated during both word reading and flanker attention tasks; (2) VWFA task-based functional connectivity increased to: (a) language network nodes, including IFG, STS, as well as attention network nodes, including FEF and IPS, during the reading task and (b) visuospatial attention network nodes during the visuospatial attention tasks. Importantly, task-based functional connectivity results show strong convergence with our structural and intrinsic functional connectivity results and are consistent with the multiplex model of VWFA function.

Supplementary Figures S1-3

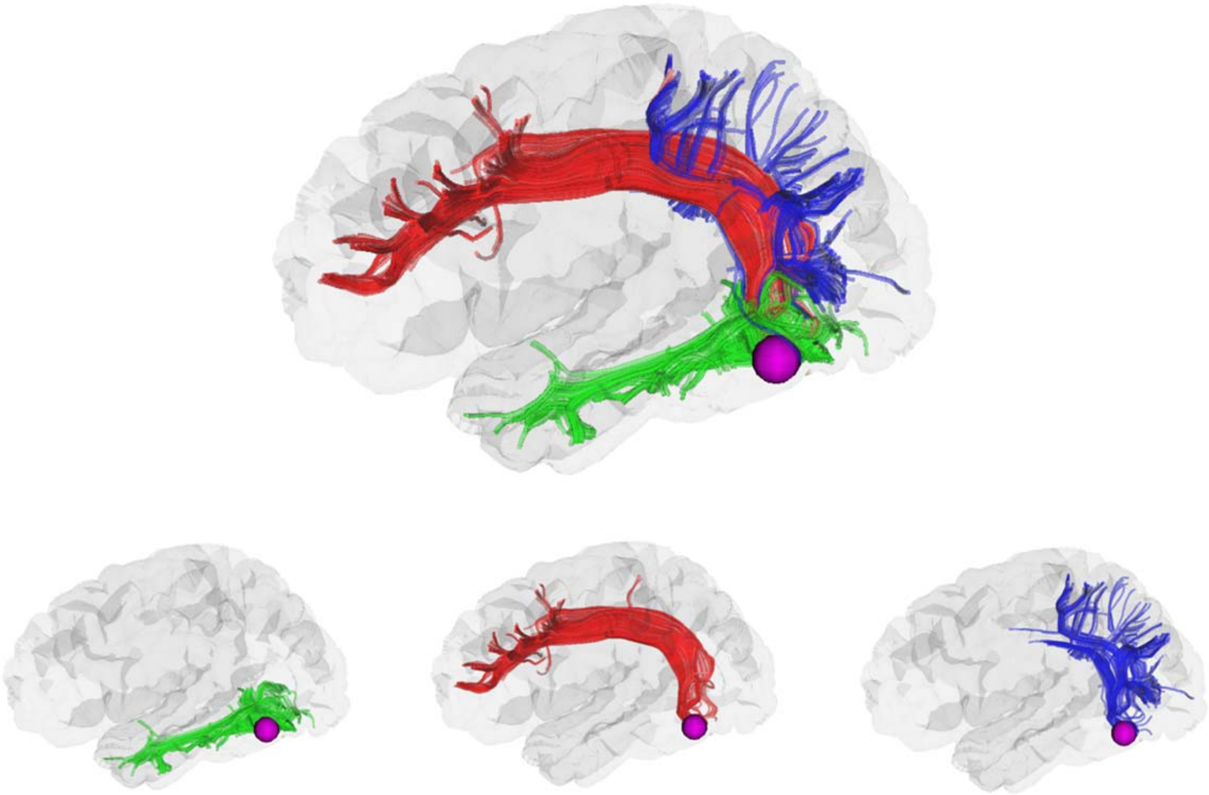

Supplementary Figure 1. Illustration of white-mater streamlined tracts from the VWFA ROI (shown in purple) in a single subject. Green: inferior longitudinal fasciculus (ILF); Red: superior longitudinal fasciculus (SLF); Blue: vertical occipital fasciculus/temporal-parietal-superior parietal lobule (vOF/TP-SPL)[16,17].

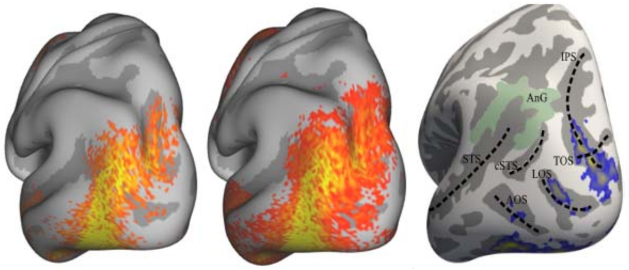

Supplementary Figure 2. Similarity between vOF/TP-SPL tracts identified in our structural connectivity analysis and the Yeatman's study[16]. *Left*: VWFA connectivity from the present study ($p<10^{-15}$). *Middle*: VWFA connectivity from the present study with a less conservative threshold ($p<10^{-5}$), which shows structural connectivity with a larger extent of the IPS. *Right*: vOF terminations reproduced from Yeatman et al. The overlap is strongest in the posterior aspects of the IPS. Importantly, our HCP-derived VWFA tracts extend more dorsally and anteriorly along the IPS than the vOF[16] consistent with TP-SPL tracts linking inferior temporal and superior parietal cortices[17].

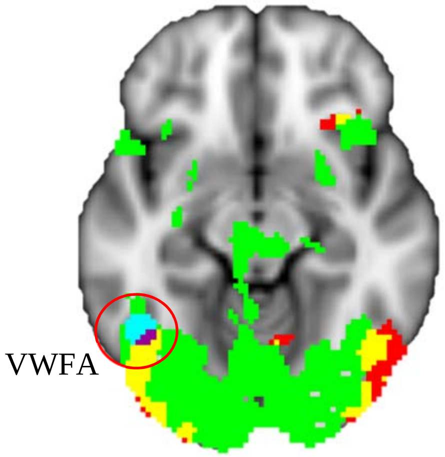

Supplementary Figure 3. Overlapping activations in VWFA for rhyming (green) and flanker (red) tasks. The binarized group-averaged activation maps were created for the reading (word vs. rest) and flanker (correct-incongruent vs. rest) tasks separately from Datasets 1 and 2, respectively (height: p <.001; cluster size = 41 voxels, p <.01). The spherical VWFA seed used in the structural and intrinsic functional connectivity analyses is plotted in cyan, and the three-way overlap of the two task activation maps and the VWFA seed is plotted in purple with peak at MNI coordinates at (-45, -57, -12). Additional overlap between these two functional tasks extended beyond VWFA (yellow), encompassing bilateral posterior fusiform gyrus (pFG).

Supplementary Tables S1-15

Supplementary Table 1. Demographic, cognitive and movement measures.

	Mean	SD	Range
**Demographic**			
Age (Years)	29.96	2.65	26 - 35
Handedness	80.99	18.27	5 - 100
**Cognitive Tests**			
Flanker	102.13	10.11	72.81 - 122.31
Word Reading	108.35	14.19	60.11 – 138.00
Picture Vocabulary	111.62	14.56	68.72 - 153.09
**Scan movement**			
Mean Displacement (mm)	0.09	0.02	0.05 - 0.19
Rotation Range X (Degree)	0.88	0.55	0.14 - 3.19
Rotation Range Y (Degree)	0.57	0.34	0.10 - 1.97
Rotation Range Z (Degree)	0.52	0.31	0.11 - 2.31
Translation Range X (mm)	0.54	0.19	0.17 - 0.99
Translation Range Y (mm)	0.34	0.17	0.10 - 0.91
Translation Range Z (mm)	0.66	0.19	0.20 - 1.00

Supplementary Table 2. Correlation matrix.

	1	2	3	4	5	6	7	8	9	10	11
1 Age	-										
2 Handedness	0.08	-									
3 Flanker	0.01	-0.04	-								
4 Word Reading	-0.16**	-0.13*	0.16**	-							
5 Picture Vocabulary	-0.18**	-0.13*	0.17**	0.66***	-						
6 Mean Displacement (mm)	0.07	0.06	-0.06	-0.2***	-0.15**	-					
7 Rotation Range X (Degree)	0.09	-0.01	-0.03	-0.02	-0.04	-0.01	-				
8 Rotation Range Y (Degree)	0.04	0.03	-0.01	0	-0.05	0.02	0.31***	-			
9 Rotation Range Z (Degree)	0.1#	-0.01	-0.04	0.03	-0.03	-0.08	0.38***	0.24***	-		
10 Translation Range x (mm)	0.16**	-0.04	0.00	-0.04	-0.03	0.34***	0.2***	0.23***	0.54***	-	
11 Translation Range y (mm)	0.08	-0.04	0.04	-0.09	-0.05	0.03	0.49***	0.25***	0.36***	0.20***	-
12 Translation Range z (mm)	0.01	0.00	-0.07	-0.12*	-0.06	0.22***	0.25***	0.21***	0.19***	0.31***	0.19***

Notes: * < .05; ** < .01; *** <.001; # <.10.

Supplementary Table 3. Beta values of structural connectivity of VWFA with target ROIs.

	Coordinates (MNI)	Mean	SD	One-sample test t-value	Effect Size (Cohen's d)
**Language ROIs**					
Vogel et al. (2012)					
Left AG	(-49,-57,28)	0.0047	0.0086	1.4336	0.0810
Left SMG	(-56,-43,31)	0.0004	0.0007	-98.1536***	-5.5480
Left IFG	(-53,27,16)	0.0011	0.0045	-11.1896***	-0.6325
Left ITG	(-61,-33,-15)	0.0082	0.0125	5.8814***	0.3324
STS ROIs from Neurosynth					
Left aSTS	(-54,9,-20)	0.0075	0.0130	4.6933***	0.2653
Left mSTS	(-53,-18,-10)	0.0224	0.0248	13.0629***	0.7384
Left pSTS	(-52,-40,5)	0.0048	0.0076	1.7982#	0.1016
**Attention ROIs**					
Vogel et al. (2012)					
Left FEF	(-26,-5,50)	0.0117	0.0189	7.2079***	0.4074
Left MT+	(-45,-71,-1)	0.1672	0.0642	44.9684***	2.5418
Left aIPS	(-25,-62,51)	0.0072	0.0111	5.0934***	0.2879
Left pIPS	(-25,-69,34)	0.0352	0.0336	16.4408***	0.9293
Swisher et al., (2007)					
IPS1		0.0156	0.0223	9.2112***	0.5207
IPS2		0.0092	0.0155	5.8661***	0.3316
IPS3		0.0045	0.0089	0.9958	0.0563
IPS4		0.0014	0.0032	-14.6578***	-0.8285
Network Comparison (Language vs. Attention)			*t* (312) = -30.54, *p* <.001		

Notes: *** suggests that the beta values of connectivity link were significant by the one-sample t test against the empirically derived median value (0.00402) at the level of $p < .001$.

Supplementary Table 4. Beta values of resting-state functional connectivity of VWFA with target ROIs.

	Coordinates (MNI)	Mean	SD	One-sample test t-value	Effect Size (Cohen's d)
**Language ROIs**					
Vogel et al. (2012)					
Left AG	(-49,-57,28)	0.0498	0.0792	10.2365***	0.5786
Left SMG	(-56,-43,31)	0.0584	0.0613	16.8520***	0.8870
Left IFG	(-53,27,16)	0.0543	0.0669	13.3027***	0.7519
Left ITG	(-61,-33,-15)	0.0248	0.0447	8.2087***	0.4640
STS ROIs from Neurosynth					
Left aSTS	(-54,9,-20)	0.0409	0.0561	11.6259***	0.6571
Left mSTS	(-53,-18,-10)	0.0494	0.0697	11.5142***	0.6508
Left pSTS	(-52,-40,5)	0.0751	0.0745	16.8660***	0.9533
**Attention ROIs**					
Vogel et al. (2012)					
Left FEF	(-26,-5,50)	0.0790	0.0541	24.4984***	1.3847
Left MT+	(-45,-71,-1)	0.1717	0.0937	31.6751***	1.7904
Left aIPS	(-25,-62,51)	0.1415	0.0785	30.9776***	1.7510
Left pIPS	(-25,-69,34)	0.1492	0.0828	31.0145***	1.7530
Swisher et al., (2007)					
IPS1		0.1351	0.0827	28.0192***	1.5837
IPS2		0.1248	0.0756	28.2508***	1.5968
IPS3		0.1246	0.0744	28.6591***	1.6199
IPS4		0.0849	0.0705	20.2942***	1.1471
Network Comparison (Language vs. Attention)				$t\,(312) = -25.03$, $p < .001$	

Notes: *** suggests that the beta values of connectivity link were significant by the one-sample t test against zero at the level of $p < .001$.

Supplementary Table 5. Beta values of structural connectivity and resting-state functional connectivity of VWFA with language ROIs based on Stevens et al. (2017).

	Coordinates (MNI)	Mean	SD	One-sample test t-value	Effect Size (Cohen's d)
**Structural Connectivity**					
Broca’s area	(-45,14.9,21.6)	0.0074	0.0124	4.8721***	0.2754
Wernicke’s area	(-49.5,-40.4,26.26)	0.0006	0.0010	-60.1241***	-3.3984
Left Postcentral	(-57.43,-11.06,45.76)	0.0005	0.0019	-32.7102***	-1.8489
**Functional Connectivity**					
Broca’s area	(-45,14.9,21.6)	0.0785	0.0674	19.5500***	1.1050
Wernicke’s area	(-49.5,-40.4,26.26)	0.0656	0.0543	20.0623***	1.1340
Left Postcentral	(-57.43,-11.06,45.76)	0.1072	0.1085	16.8301***	0.9513

Notes: *** suggests that the beta values of connectivity link were significant by the one-sample t test at the level of $p < .001$.

Supplementary Table 6. Beta values of structural connectivity and resting-state functional connectivity of VWFA with ROIs in anterior temporal lobe (ATL).

	Coordinates (MNI)	Mean	SD	One-sample test t-value	Effect Size (Cohen's d)
**Structural Connectivity**					
ATL (Woollams et al., 2017)	(-53,13,-32)	0.0215	0.0247	12.5582***	0.7098
sATL (Hoffman et al., 2015)	(-54,6,-22)	0.0168	0.0212	10.6502***	0.6020
vATL (Hoffman et al., 2015)	(-42,-14,-34)	0.0714	0.0468	25.4349***	1.4377
vATL (Visser et al., 2011)	(-44,-38,40)	0.3264	0.0924	61.6977***	3.4874
**Functional Connectivity**					
ATL (Woollams et al., 2017)	(-53,13,-32)	0.0277	0.0491	8.5279***	0.4820
sATL (Hoffman et al., 2015)	(-54,6,-22)	0.0420	0.0592	11.3337***	0.6406
vATL (Hoffman et al., 2015)	(-42,-14,-34)	0.0295	0.0413	10.9207***	0.6173
vATL (Visser et al., 2011)	(-44,-38,40)	0.3919	0.0767	89.4633***	5.0568

Notes: *** suggests that the beta values of connectivity link were significant by the one-sample t test at the level of $p < .001$.

Supplementary Table 7. Comparison of beta values of structural connectivity and resting-state functional connectivity of VWFA with two SMG ROIs.

	Coordinates (MNI)	Mean	SD	One-sample test t-value	Effect Size (Cohen's d)
**Structural Connectivity**					
SMG (Vogel et al, 2012)	(-56,-43,31)	0.0004	0.0007	-98.1536***	-5.5480
SMG (Neurosynth)	(-44,-38,40)	0.0038	0.0073	-0.4729	-0.0267
**Functional Connectivity**					
SMG (Vogel et al, 2012)	(-56,-43,31)	0.0584	0.0613	16.8520***	0.8870
SMG (Neurosynth)	(-44,-38,40)	0.1045	0.0734	24.2318***	1.3697

Notes: *** suggests that the beta values of connectivity link were significant by the one-sample t test at the level of $p < .001$.

Supplementary Table 8. Multiple regression analysis of VWFA structural connectivity predicting age-adjusted Picture Vocabulary scores.

	Beta value	Std. Error	t-value	p-value
Intercept	109.096	1.553	70.235	< .001***
Gender (F=0)	4.98	1.672	2.979	0.003**
Functional Connectivity (FC)				
Left aSTS	15.172	22.238	0.682	0.496
Left mSTS	9.831	19.076	0.515	0.607
Left pSTS	-13.227	19.44	-0.68	0.497
Structural Connectivity (SC)				
Left aSTS	144.659	71.278	2.03	0.043*
Left mSTS	-95.006	41.792	-2.273	0.024*
Left pSTS	306.86	121.845	2.518	0.012*

Model Comparison	R-square	F-value	Effect Size	Bayes Factor
1 Baseline (1 + Gender)	0.037	11.810***	0.038	33.106
2 Functional (1 + Gender + FC)	0.039	3.130*	0.041	0.279
3 Structural (1 + Gender + SC)	0.068	5.556***	0.072	**20.859**
4 Full (1 + Gender + FC + SC)	0.071	3.299**	0.076	0.717

Notes: * < .05; ** < .01; *** <.001; # <.10. Bolded numbers highlighted the large Bayes Factor for detecting a small effect.

Supplementary Table 9. Multiple regression analysis of VWFA structural connectivity predicting age-adjusted Word Reading scores.

	Beta value	Std. Error	t-value	p-value
Intercept	106.835	1.529	69.876	< .001***
Gender (F=0)	3.672	1.646	2.231	0.026*
Functional Connectivity (FC)				
Left aSTS	-8.593	21.889	-0.393	0.695
Left mSTS	14.727	18.776	0.784	0.434
Left pSTS	4.613	19.135	0.241	0.810
Structural Connectivity (SC)				
Left aSTS	62.346	70.159	0.889	0.375
Left mSTS	-104.26	41.136	-2.535	0.012*
Left pSTS	250.101	119.932	2.085	0.038*

Model Comparison	R-square	F-value	Effect Size	Bayes Factor
1 Baseline (1 + Gender)	0.021	6.805**	0.022	3.198
2 Functional (1 + Gender + FC)	0.028	2.174#	0.028	0.044
3 Structural (1 + Gender + SC)	0.046	3.716**	0.048	0.721
4 Full (1 + Gender + FC + SC)	0.051	2.345*	0.053	0.036

Notes: * < .05; ** < .01; *** <.001; # <.10.

Supplementary Table 10. Multiple regression analysis of VWFA structural connectivity predicting age-adjusted Flanker score.

	Beta value	Std. Error	t-value	p-value
Intercept	95.425	2.204	43.303	<.001***
Gender (F=0)	3.068	1.163	2.638	0.008**
Functional Connectivity (FC)				
Left FEF	15.024	14.586	1.03	0.74348
Left MT+	2.91	8.884	0.328	0.41018
Left aIPS	9.793	11.874	0.825	0.75599
Left pIPS	-3.141	10.097	-0.311	0.74348
Structural Connectivity (SC)				
Left FEF	-106.055	32.402	-3.273	0.001**
Left MT+	20.815	9.465	2.199	0.028*
Left aIPS	-95.559	60.567	-1.578	0.116
Left pIPS	38.406	19.694	1.95	0.052#

Model Comparison	R-square	F-value	Effect Size	Bayes Factor
1 Baseline (1 + Gender)	0.016	3.645#	0.012	0.720
2 Functional (1 + Gender + FC)	0.021	1.307	0.021	0.004
3 Structural (1 + Gender + SC)	0.070	4.619***	0.075	**5.731**
4 Full (1 + Gender + FC + SC)	0.090	3.324***	0.099	0.886

Notes: * < .05; ** < .01; *** <.001; # <.10. Bolded numbers highlighted the large Bayes Factor for detecting a small effect.

Supplementary Table 11. Multiple regression analysis of VWFA intrinsic functional connectivity predicting age-adjusted Picture Vocabulary score.

	Beta value	Std. Error	t-value	p-value
Intercept	115.092	6.104	18.856	<.001***
Gender (F=0)	4.95	1.728	2.865	0.004**
Functional Connectivity (FC)				
ATL (Woollams et al., 2017)	-63.194	26.441	-2.39	0.017*
sATL (Hoffman et al., 2015)	60.109	22.532	2.668	0.008**
vATL (Hoffman et al., 2015)	7.159	24.255	0.295	0.768
vATL (Visser et al., 2011)	-19.582	13.169	-1.487	0.138
Structural Connectivity (SC)				
ATL (Woollams et al., 2017)	21.662	46.1	0.47	0.639
sATL (Hoffman et al., 2015)	24.292	51.868	0.468	0.640
vATL (Hoffman et al., 2015)	11.519	20.683	0.557	0.578
vATL (Visser et al., 2011)	-1.358	9.179	-0.148	0.882

Model Comparison	R-square	F-value	Effect Size	Bayes Factor
1 Baseline (1 + Gender)	0.037	11.810***	0.038	33.106
2 Functional (1 + Gender + FC)	0.0637	4.162**	0.068	**2.896**
3 Structural (1 + Gender + SC)	0.0437	2.794*	0.046	0.147
4 Full (1 + Gender + FC + SC)	0.0714	2.578**	0.077	0.097

Notes: * < .05; ** < .01; *** <.001; # <.10. Bolded numbers highlighted the large Bayes Factor for detecting a small effect.

Supplementary Table 12. VWFA functional connectivity with language and attention network nodes during the rhyming task.

Target ROIs	Mean (SD)	t-value	Uncoorr. p-value	FDR adjusted p-value
**Language ROIs**				
Vogel et al. 2012				
Left IFG	0.31 (0.36)	3.09	0.009	0.017*
Left ITG	0.11 (0.39)	1.04	0.320	0.358
Left AG	0.09 (0.33)	1.03	0.325	0.358
Left SMG	0.26 (0.24)	3.92	0.002	0.007**
STS ROIs from Neurosynth				
Left aSTS	0.09 (0.37)	0.87	0.401	0.401
Left mSTS	0.28 (0.35)	2.92	0.013	0.020*
Left pSTS	0.39 (0.27)	5.14	<.001	0.001**
**Attention ROIs**				
Vogel et al. 2012				
Left FEF	0.21 (0.33)	2.35	0.037	0.051#
Left MT+	0.32 (0.32)	3.61	0.004	0.008**
Left aIPS	0.29 (0.19)	5.58	<.001	0.001**
Left pIPS	0.35 (0.34)	3.78	0.003	0.007**

Notes: * < .05; ** < .01; *** <.001; # <.10 for the FDR corrected p values.

Supplementary Table 13. VWFA functional connectivity with language and attention network nodes during the flanker task.

Target ROIs	Mean (SD)	t-value	Uncoorr. p-value	FDR adjusted p-value
**Language ROIs**				
Vogel et al. 2012				
Left IFG	0.19 (0.35)	2.75	0.011	0.019*
Left ITG	0.08 (0.46)	0.9	0.379	0.379
Left AG	0.10 (0.45)	1.18	0.25	0.274
Left SMG	0.34 (0.48)	3.61	0.001	0.003**
STS ROIs from Neurosynth				
Left aSTS	0.21 (0.4)	2.7	0.012	0.019*
Left mSTS	0.17 (0.37)	2.39	0.025	0.034*
Left pSTS	0.18 (0.43)	2.09	0.047	0.057#
**Attention ROIs**				
Vogel et al. 2012				
Left FEF	0.34 (0.41)	4.19	<.001	0.001**
Left MTplus	0.32 (0.39)	4.2	<.001	0.001**
Left aIPS	0.43 (0.43)	5.07	<.001	<.001***
Left pIPS	0.37 (0.27)	7.07	<.001	<.001***

Notes: * < .05; ** < .01; *** <.001; # <.10 for the FDR corrected p values.

Supplementary Table 14. Comparison of the GAM and GLM results for the brain-behavior relationship.

	Additional R^2 explained by GAM	Chi-square value (deviance test of GAM over GLM)
Picture Vocabulary	0.017	9.639*
Word Reading	0.002	3.680
Flanker Task	0.068	43.798**

Notes: * <.05; ** <.01. GAM = General Additive Model; GLM = General Linear Model. Compared to the GLM, the nonparametric GAM analysis explained an additional 1.7% ~ 6.8% variance on the Picture Vocabulary and Flanker Tasks.

Supplementary Table 15. Spearman correlation of predicted scores from structural connectivity and actual scores of behavioral measures.

	Picture Vocabulary	Word Reading	Flanker Task
VWFA-STS circuitry	**0.16****	**0.16****	0.01
VWFA-frontoparietal circuitry	0.10#	0.05	**0.20*****

Notes: ** <.01, ***<.001, #<.10.

Supplementary References

1. Mennes, M. *et al.* Inter-individual differences in resting-state functional connectivity predict task-induced BOLD activity. *Neuroimage* **50**, 1690–1701 (2010).

2. Mennes, M. *et al.* Linking inter-individual differences in neural activation and behavior to intrinsic brain dynamics. *Neuroimage* **54**, 2950–2959 (2011).

3. Clare Kelly, A. M., Uddin, L. Q., Biswal, B. B., Castellanos, F. X. & Milham, M. P. Competition between functional brain networks mediates behavioral variability. *Neuroimage* **39**, 527–537 (2008).

4. Ashburner, J. *et al.* SPM12 Manual （The FIL Methods Group ）. *Funct. Imaging Lab.* (2013). doi:10.1111/j.1365-294X.2006.02813.x

5. Eklund, A., Nichols, T. E. & Knutsson, H. Cluster failure: Why fMRI inferences for spatial extent have inflated false-positive rates. *Proc. Natl. Acad. Sci. U. S. A.* (2016). doi:10.1073/pnas.1602413113

6. McLaren, D. G., Ries, M. L., Xu, G. & Johnson, S. C. A generalized form of context-dependent psychophysiological interactions (gPPI): A comparison to standard approaches. *Neuroimage* (2012). doi:10.1016/j.neuroimage.2012.03.068

7. Binder, J. R., Desai, R. H., Graves, W. W. & Conant, L. L. Where Is the Semantic System? A Critical Review and Meta-Analysis of 120 Functional Neuroimaging Studies. *Cereb. Cortex* **19**, 2767–2796 (2009).

8. Stevens, W. D., Kravitz, D. J., Peng, C. S., Henry Tessler, M. & Martin, A. Privileged Functional Connectivity Between the Visual Word Form Area and the Language System. *J. Neurosci.* **37**, 0138–17 (2017).

9. McCandliss, B. D., Cohen, L. & Dehaene, S. The visual word form area: Expertise for reading in the fusiform gyrus. *Trends Cogn. Sci.* **7**, 293–299 (2003).

10. Swisher, J. D., Halko, M. A., Merabet, L. B., McMains, S. A. & Somers, D. C. Visual Topography of Human Intraparietal Sulcus. *J. Neurosci.* **27**, 5326–5337 (2007).

11. Vogel, A. C., Miezin, F. M., Petersen, S. E. & Schlaggar, B. L. The putative visual word form area is functionally connected to the dorsal attention network. *Cereb. Cortex* **22**, 537–549 (2012).

12. Gao, W. & Lin, W. Frontal Parietal Control Nework Regulates the Anti-Correlated Default and Dorsal Attention Networks. *Hum. Brain Mapp.* **33**, 192–202 (2012).

13. Xue, G., Chen, C., Jin, Z. & Dong, Q. Language experience shapes fusiform activation when processing a logographic artificial language: an fMRI training study. *Neuroimage* **31**, 1315–1326 (2006).

14. Price, C. J. & Devlin, J. T. The myth of the visual word form area. *Neuroimage* **19**, 473–481 (2003).

15. Dundas, E. M., Plaut, D. C. & Behrmann, M. The joint development of hemispheric

lateralization for words and faces. *J. Exp. Psychol. Gen.* **142**, 348–358 (2013).

16. Yeatman, J. D. *et al.* The vertical occipital fasciculus: A century of controversy resolved by in vivo measurements. *Proc. Natl. Acad. Sci.* 201418503 (2014). doi:10.1073/pnas.1418503111

17. Bullock, D. *et al.* Associative white matter connecting the dorsal and ventral posterior human cortex. *Brain Struct. Funct.* **224**, 2631–2660 (2019).
